# Sequential and iterative Pd-catalyzed cross-coupling reactions in organic synthesis

**DOI:** 10.1007/s00706-016-1883-7

**Published:** 2016-12-09

**Authors:** Patrick Dobrounig, Melanie Trobe, Rolf Breinbauer

**Affiliations:** Institute of Organic Chemistry, Graz University of Technology, Graz, Austria

**Keywords:** Biaryls, Boronic acid, Heterocycles, Leaving group, Ligand, Palladium, Suzuki coupling

## Abstract

**Abstract:**

Sequential and iterative Pd-catalyzed cross-coupling reactions can be performed in which the order of C–C bond formations can be controlled either by the attenuated leaving groups of the multireactive substrate or by specific catalyst/ligand combinations. This tutorial review gives an overview about recent developments in this field and the various strategies used for the assembly of oligoarenes and -alkenes.

**Graphical abstract:**

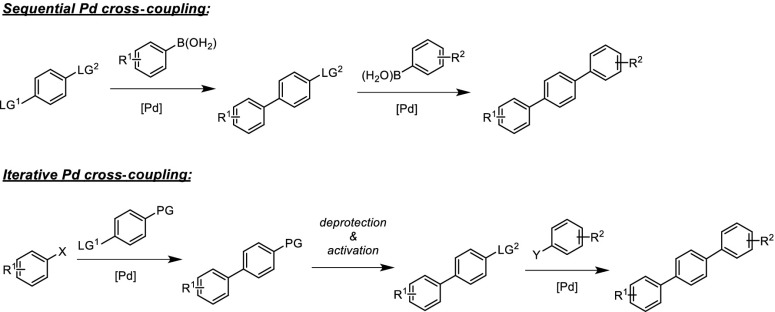

## Introduction

The development of Pd-catalyzed cross-coupling reactions had a tremendous influence on organic synthesis. These reactions not only have enriched our methodology tool box but also inspired the design of a new generation of drugs and materials. In the synthesis of such materials, not only one, but quite often two or even more cross-coupling reactions are used [1]. As a consequence, strategies have been developed, which allow the use of Pd-catalyzed reactions in a sequential or iterative way. The latter case involves an intermediate deprotection step which makes the leaving group suitable for another coupling step (Scheme 1).

**Figure:**
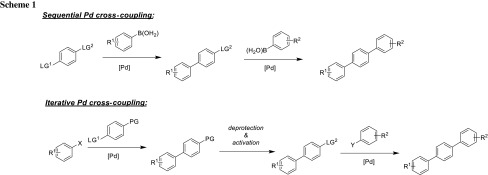

Several options exist to achieve selectivity in these coupling sequences: (1) through attenuated reactivity of the leaving groups at the electrophilic substrate, (2) through differentiated reactivity of nucleophilic substrate, and (3) by catalyst control. In this review, we present an overview about the state-of-the-art of this field with a special focus on the most recent developments since 2009.

## Regio- and chemoselectivity at the electrophilic coupling partner

### Electrophiles with two halogens/pseudohalogens differing in their reactivity

The reactivity of the electrophilic substrate in Pd-catalyzed reactions depends highly on its leaving group, which decreases in the following order –N_2_^+^ > –I > –Br ~ -OTf > –Cl. Choosing a highly reactive electrophile leads on the one hand most likely to very satisfactory cross-coupling results at mild reaction conditions with conventional Pd-catalysts [e.g. Pd(PPh_3_)_4_], but on the other hand, stability, compatibility with the previous steps, and availability of these reactive compounds may be a major concern. Compared to their chloro-substituted homologs, fewer iodinated compounds are commercially available and only at higher cost. Therefore, considerable effort has been invested to identify conditions under which these less reactive chlorides undergo effective cross-coupling. For this purpose, Buchwald and coworkers have developed a series of sterically demanding phosphine ligands, which allow cross-coupling reactions with chlorides at rather mild reaction conditions [2, 3].

The differentiated reactivity of these leaving groups can be exploited for sequential cross-coupling. The more reactive leaving group should undergo chemoselective cross-coupling in the first reaction followed by the less reactive one in a second cross-coupling step. The group of Bazin synthesized a 1,6-naphthyridin-2(1*H*)-one (**3**) library through sequential cross-coupling. When they investigated the reaction conditions (Scheme 2; Table 1), they observed that the bromide and chloride functions differ insufficiently under their reaction conditions to completely exclude double cross-coupling (Table 1, entries 1–4). Switching the leaving group from bromide to iodide gave the desired outcome in even short reaction time (Table 1, entry 5). Under microwave irradiation, the reaction time could be further decreased, but with a loss in yield (Table 1, entry 6) [4].

**Figure:**
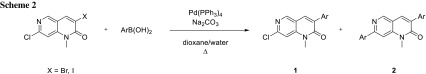

**Table 1:** Chemoselective Suzuki–Miyaura cross-coupling of 1,6-naphthyridin-2(1*H*)-ones [4]

Entry	X	ArB(OH)_2_	*T*/°C	Time	**1**/%^a^	**2**/%^a^
1	Br		105	3 h	67	27
2	Br		105	2 h	68	9
3	Br		105	1 h	52	Traces
4	Br		80	4 h	74	Traces
5	I		80	3 h	74	–
6^b^	I		80	10 min	67	–

Reagents and conditions: substrate (0.3 mmol), ArB(OH)_2_ (1.1 equiv.), Pd(PPh_3_)_4_ (5 mol%), Na_2_CO_3_ (2.5 equiv.), 1,4-dioxane/water (4:1, 5 cm^3^), 80–105 °C, sealed vessel

^a^Isolated yield after purification

^b^Reaction performed in a sealed vessel in a microwave reactor (fixed temperature and variable pressure)

For the second step, microwave heating resulted in complete conversion in 2 h. As they use the same catalyst in both couplings, they tried to do both in a one-pot procedure by adding more catalyst, base, and the second boronic acid after the first coupling (Scheme 3; Table 2). This one-pot procedure proved to be very convenient resulting in higher yields (Table 2, entries 1, 2) [4].

**Figure:**
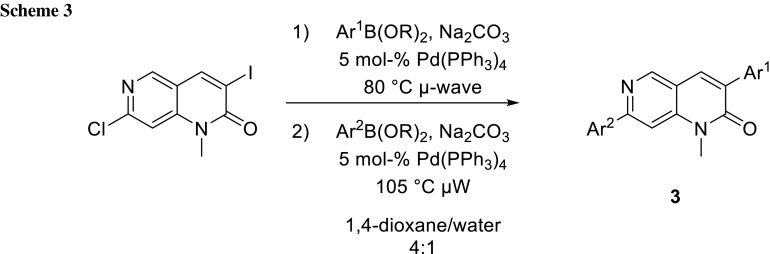

**Table 2:** One-pot procedure for the synthesis of 1,6-naphthyridin-2(1*H*)-ones [4]

Entry	Ar^1^B(OR)_2_	Time	Ar^2^B(OR)_2_	Time/h	Yield/%^a^
1^b^		3 h		2	51
2		10 min		3	58
3		10 min		2	55
4		1.25 h		1.5	32
5		1.5 h		3.5	33

^a^Isolated yield after purification

^b^Monocoupled product was isolated before the second step

A very widely used combination is iodide and bromide [5–8]. Stanetty and coworkers recognized that the 5-position in thiazoles is the most activated position for oxidative addition [9], which attenuates even further the reactivity difference between iodide and bromide in their system (**4**), which allows them to perform chemoselective cross-coupling reactions although using the very same conditions in both steps (Scheme 4) [6].

**Figure:**
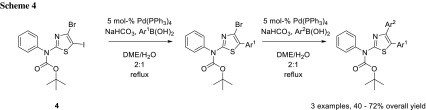

The group of Takahashi also utilized the different reactivity of iodide and bromide to synthesize a library of ligands (**5**, Scheme 5). For the first coupling step, they used Pd(PPh_3_)_4_ as a catalyst and for the second cross-coupling Pd_2_(dba)_3_ with the bulky and electron rich phosphine ligand *t*Bu_3_P. With this catalyst system, they could also achieve double coupled products (**6**) in a single step [5].

**Figure:**
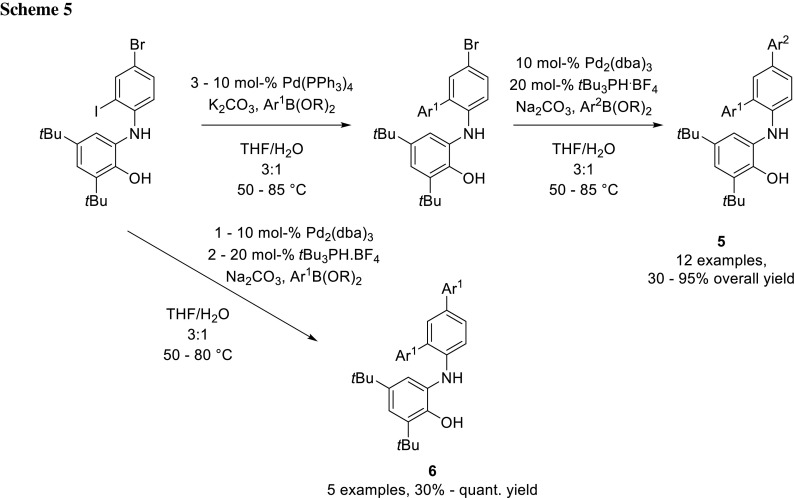

The group of Abbotto and Almqvist utilized the difference in reactivity between iodide and bromide for the synthesis of substituted pyridine derivatives (**7**) and the group of Almqvist for 2,3-dihydro-5*H*-thiazolo[3,2-*a*]pyridin-5-one derivatives (**8**), respectively (Scheme 6) [7, 8].

**Figure:**
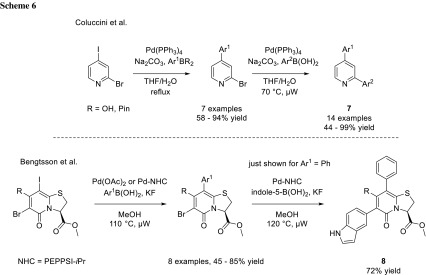

Breinbauer and coworkers also used sequential cross-coupling for their modular synthesis of teraryl-based α-helix mimetics (**9**) [10–13]. In their earlier work, they utilized the highly reactive diazonium group to do the first cross-coupling and the less reactive bromide for the second one (Scheme 7) [11]. Later, they changed their strategy using triflate and iodide as leaving groups, which simplified the synthesis of the respective building blocks (Scheme 8) [10–13].

**Figure:**
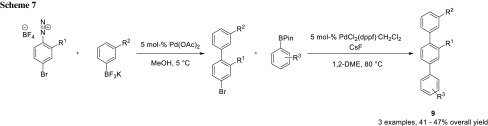

**Figure:**
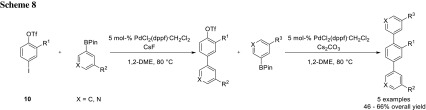

PdCl_2_(dppf)·CH_2_Cl_2_ proved to be the best Pd precatalyst for both coupling steps. The iodide was selectively coupled first with CsF as the base, whereas triflate was coupled with Cs_2_CO_3_ in the second step. They also attempted a one-pot reaction, by just adding Cs_2_CO_3_ and the second boronic ester after complete conversion of the first coupling reaction. While the one-pot procedure worked for some examples, for others, homocoupling and various side reactions became an issue; therefore, the two-step reaction is recommended as a standard procedure [11]. If a twofold coupling with the same boronic ester is desired, this is possible using the conditions of the second coupling with a higher Pd loading [12].

The combination of bromide and chloride shows a large intrinsic reactivity difference in sequential cross-coupling. Haley and coworkers coupled selectively the bromide under standard cross-coupling conditions, and by switching, the ligand to SPHOS they transformed the chloride in a second cross-coupling step (Scheme 9) [14].

**Figure:**
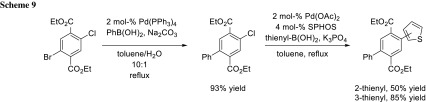

The difference in reactivity between iodide and chloride is even higher. Manetsch and coworkers have shown in their work that this motif worked smoothly for sequential cross-coupling (Scheme 10). Interestingly, they could use the same reaction conditions for both steps [15].

**Figure:**
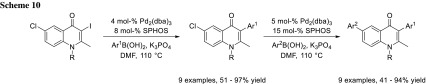

A more unconventional orthogonal leaving group pair is iodide and the thiomethyl group. The latter one can be selectively coupled with boronic acids using Pd-catalyst with Cu(I)-thiophen-2-carboxylic acid (CuTC) as a reagent under base-free conditions (Liebeskind-Srogl method) [16–19]. The group of Mihovilovic used this approach to synthesize a library of 2,3-substituted pyridines (**11**) (Scheme 11) [20].

**Figure:**
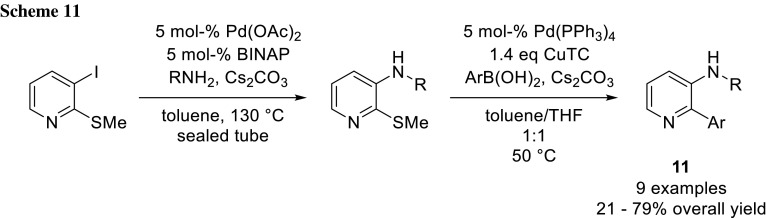

They further investigated the possibility of a one-pot procedure for this cross-coupling sequence by simply adding CuTC and the second boronic acid after the completion of the first amination step. That procedure resulted in no conversion, only when they also added additional Pd-catalyst, 21% conversion was detected after 24 h. Therefore, they developed a rapid work-up procedure, and with the resulting crude product, the second coupling step worked smoothly [20].

Theoretically, it should be possible to reverse the coupling sequence, as there is no base needed for the Liebeskind–Srogl method. The authors tested this hypothesis, but only coupling at the 3-position was detected. A possible reason for this is that the pyridine itself is sufficiently basic for Suzuki–Miyaura coupling [20].

The groups of Muller and Balme took advantage of the difference in reactivity of iodide and bromide to perform sequential cross-coupling using a one-pot procedure, by simply adding the second boronic acid after the first reaction step and rising the temperature for the second cross-coupling (Scheme 12) [21, 22].

**Figure:**
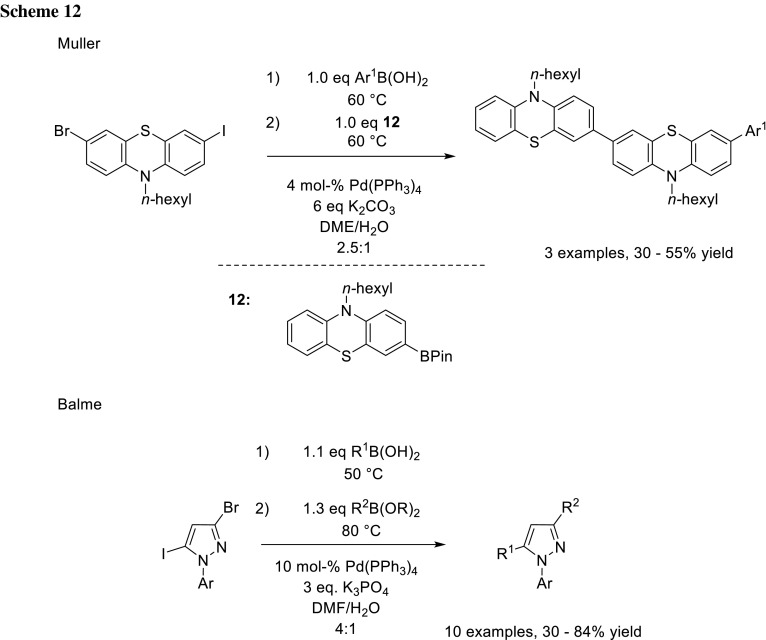

### Inversion of chemoselectivity by reagent control

While the examples given in the previous section have relied on the intrinsic reactivity differences of the leaving groups, recent developments show that it is even possible to differentiate between leaving groups which are closely related in reactivity, by activating them specifically with very defined reaction conditions. By changing conditions (catalyst, ligand system, and/or additives), the chemoselectivity can be inversed. Hayashi’s group has shown this for bromide and triflate and Fu’s group for triflate and chloride (Scheme 13; Table 3) [23, 24].

**Figure:**
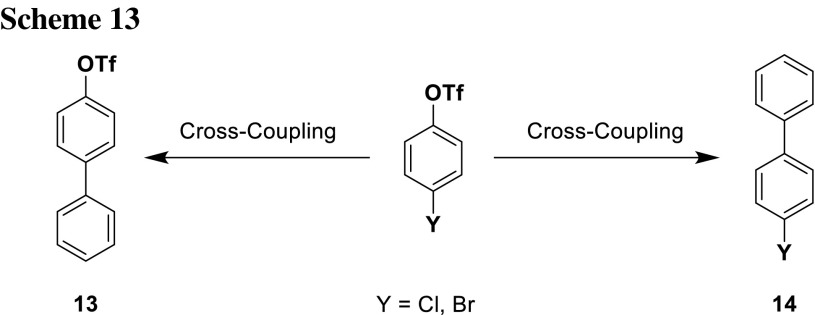

**Table 3:** Different chemoselectivity under various conditions

Entry	Y	Catalyst	Additive	Solvent	*T*/°C	Yield/%
1 [23]	Br	PdCl_2_(dppp)	LiBr	Et_2_O	0	97 (**14**)
2 [23]	Br	PdCl_2_(MeO–MOP)_2_	–	Et_2_O	20	68 (**13**)
3 [24]	Cl	Pd(OAc)_2_/PCy_3_	KF	THF	RT	87 (**14**)
4 [24]	Cl	Pd_2_(dba)_3_/P^*t*^Bu_3_	KF	THF	RT	95 (**13**)

These results indicate that there is much more behind the mechanism of oxidative addition than simple reactivity of leaving groups. According to their bond dissociation energies (BDE), the Ar–Cl (BDE = 379.3 kJ/mol [25, 26]) should be easier cleavable than Ar-OTf (BDE = 425.0 kJ/mol) [26]. Inspired by the experimental results of Fu’s group, Schönebeck and coworkers further investigated theoretically and experimentally the mechanism behind this inversion of chemoselectivity. Theoretical calculations resulted that in Fu’s system, Pd is monoligated (PdL) when using P*t*Bu_3_ as a ligand (Table 3, entry 4). With PdL, the transition state of Ar–Cl cleavage lies lower in energy than Ar-OTf. When using PCy_3_ as ligand, Pd is bisligated (PdL_2_) (Table 3, entry 3). PdL_2_ stabilizes the transition state of Ar-OTf cleavage, which explains the reaction outcome [26].

The group of Schönebeck further investigated the influence of solvent and additives on the chemoselectivity with Fu’s catalyst system (Pd_2_(dba)_3_/P*t*Bu_3_). In the nonpolar solvent toluene, Ar–Cl insertion exclusively took place in analogy to Fu’s example in THF. By changing the solvent to polar solvents like DMF and MeCN, the selectivity was reversed predominantly resulting in Ar-OTf insertion. The hypothesis that solvent coordination would result in higher ligated Pd-species was discounted by in silico calculations, which suggest that the catalytically active species in polar solvent may be a Pd ate-complex ([Pd(P*t*Bu_3_)(X)]^−^, X=F^−^, RBO_2_H^−^). The calculation corroborated this hypothesis and for their experimental proof they choose the Stille coupling (Scheme 14) as stannanes are not coordinating and the cross-coupling also works under base-free conditions. To exclude effects of attendant cations, they added the noncoordinating KPF_6_ salt to their experiments without F^−^. Their experiments and computational calculations provided a strong support for their mechanistic proposal [27, 28].

**Figure:**
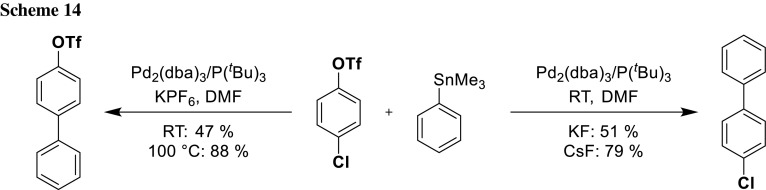

### Controlled regioselectivity through the molecule’s electronic and steric properties (substrate control)

In many cases, it is not necessary to have two different halides/pseudohalides to achieve sequential cross-coupling. Sometimes, two identical halides/pseudohalogenides exhibit different reactivity through their position in the molecule. Electronically, sterically, and/or coordinating effects can lead to regioselective cross-coupling. The influence of these effects will be discussed in the following section.

Mihovilovic and coworkers showed in their work that 2,3-dichloropyridines (**15**) undergo Buchwald–Hartwig amination selectively at the more electron-deficient 2-position. By changing the ligand to the bulky XPhos ligand, they achieved coupling at the less activated 3-position subsequently in a Suzuki–Miyaura coupling (Scheme 15) [20].

**Figure:**
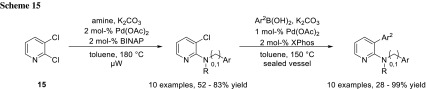

Langer’s group utilized the unequal electron-density distribution in 3,4-bis(trifluoromethylsulfonoxy)benzophenone (**16**) to synthesize a library of 3,4-diarylbenzophenones. In the first coupling reaction, the more electron-deficient carbon in *para*-position to the electron-withdrawing carbonyl group reacted regioselectively (Scheme 16). Surprisingly, they have not reported of any double-arylation, although they have used an excess of boronic acid (1.3 eq.) in the first coupling step with the very same conditions as in the second coupling step [29].

**Figure:**
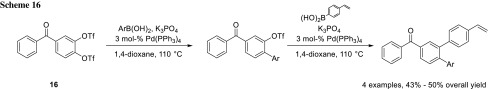

Ohta and coworkers used 2,4,5-tribromoimidazole (**17**) as starting material for their total synthesis of nortopsentin D. In the first step, they coupled selectively at the 2-position. Despite the similar reactivity of the 4- and 5-position, they achieved selective coupling at the 5-position in acceptable yields. Probably, the directing properties of MEM and SEM are sufficient to distinguish between these positions (Scheme 17) [30]. The group of Mihovilovic also used this substrate for their synthesis of neurodazines. For the second cross-coupling with the benzyl-protected imidazole, they observed a maximum selectivity of 2:1 in favor of the 2,5-diaryl over 2,4,5-triaryl product. The steric effect and the negligible electronic influence of the benzyl group are accounted for the poor selectivity. They developed a one-pot procedure, in which they couple at the 2-position first with the first boronic acid and, subsequently, the 4- and 5-position by simply adding the second boronic acid (Scheme 17) [31].

**Figure:**
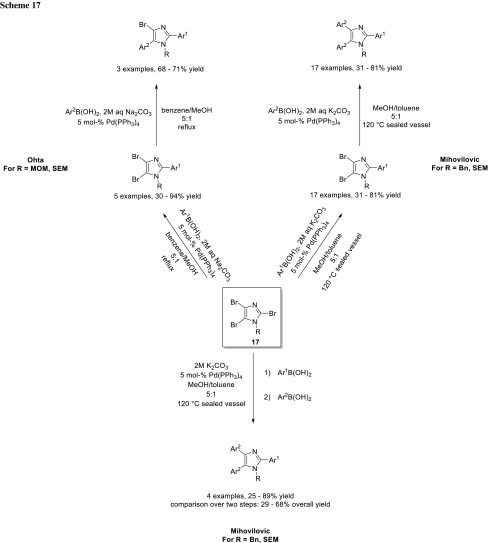

In the total synthesis of Vialinin B by Takahashi’s group as well as in the total synthesis of boletopsins 7, 11, and 12 by Barrow’s group, the two bromides in **18** were coupled in a sequential fashion. Both groups used the same substrate and coupled selectively the bromide in *ortho*-position to the carbaldehyde in the first coupling step (Scheme 18). The electronic influence of the substituents was sufficient to couple selectively the more electron-deficient position first [32, 33].

**Figure:**
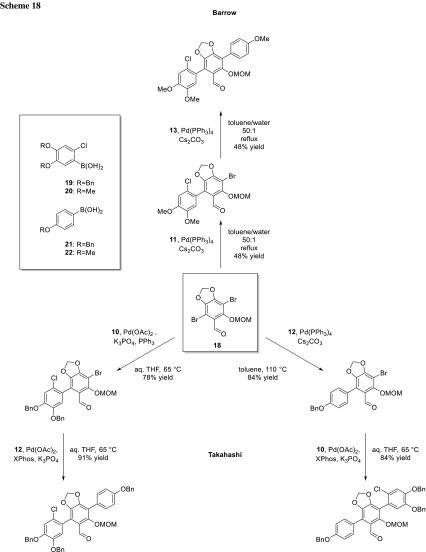

The group of Manabe developed an interesting ligand system, with which it is possible to change the regioselectivity of phenols and anilines using a different ligand (Schemes 19, 20) [34–37]. The *ortho*-selectivity using Ph-HTP as ligand can be explained through the coordination of the deprotonated phenol moiety of the ligand as well as the substrate to a Mg ion, which guides the Pd direct to the *ortho*-bromide (**25**) (Scheme 20) [34–36]. When other ligands are used, the steric environment leads to cross-coupling at the more accessible position (Scheme 21) [34–37].

**Figure:**
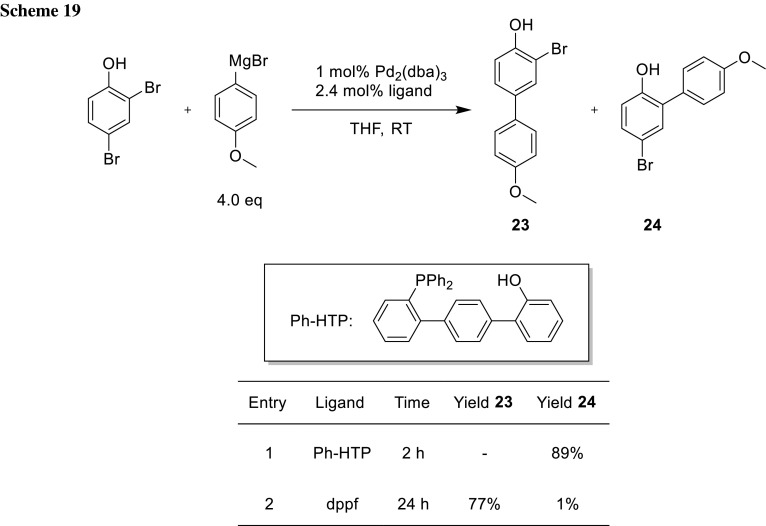

**Figure:**
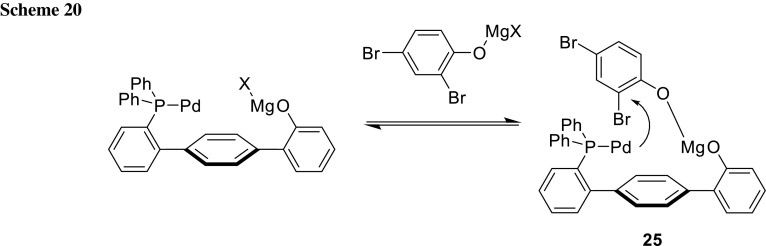

**Figure:**
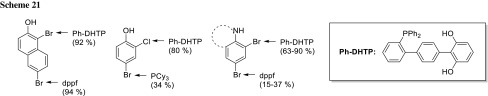

Manabe and coworkers further utilized their system to synthesize substituted benzo[*b*]furans (**26**) and indoles (**27**) from dichlorophenols and dichloroanilines, respectively, in a sequential manner. They changed their conditions by deprotonating the phenolic functions with *t*BuOLi first for enabling them to coordination. The first step is an *ortho*-selective Sonogashira reaction with subsequent cyclization and finally with a second cross-coupling step in one-pot (Scheme 22) [37–39].

**Figure:**
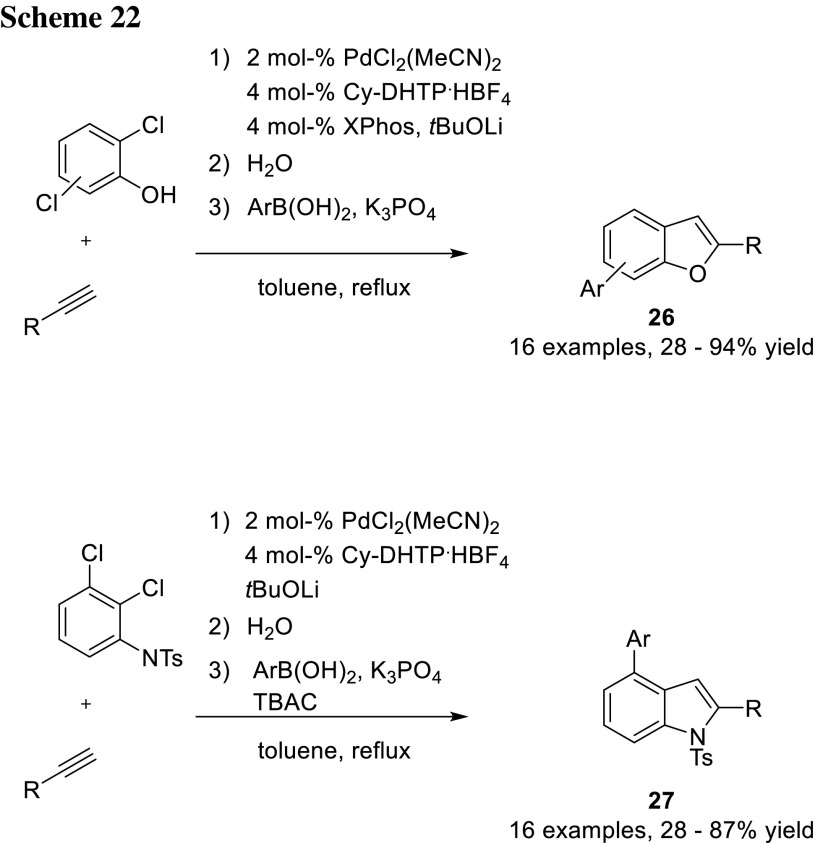

### Sequential cross-coupling of geminal dibromoalkenes

The strategy of sequential cross-couplings is not only limited to arene substrates, but can also be extended to alkene scaffolds. Carpita and coworkers realized that the olefin geometry of bromoalkenes has a huge impact on the kinetics of Suzuki–Miyaura cross-coupling. Their experiments showed that *E*-isomers react much faster than the corresponding *Z*-isomers [40]. Brown’s group utilized these results to investigate reaction conditions to stereoselectively couple the *E*-Br of geminal dibromoalkenes with boronic acids (Scheme 23) [41]. The group of Brückner used a similar set-up for their synthesis of polyunsaturated butenolides (**30**). Their experiments resulted in good stereoselectivity with very small amounts of the other isomer (Scheme 23) [42].

**Figure:**
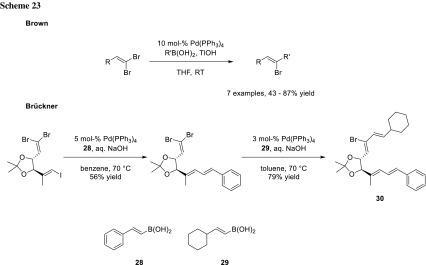

For their strategy towards the total synthesis of apoptolidin, Sulikowski and coworkers utilized the stereoselective cross-coupling of geminal dibromoalkenes. After their first coupling step, they transformed the resulting product (**31**) into a new geminal dibromoalkene (**32**) via a Corey–Fuchs reaction, which was subsequently subjected again to a stereoselective coupling (Scheme 24) [43].

**Figure:**
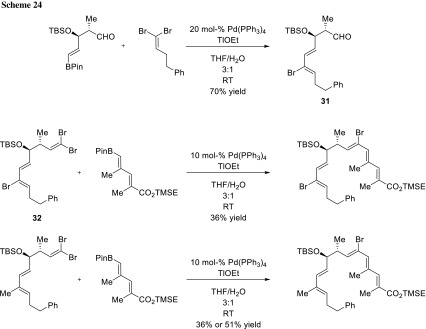

The previous examples showed the possibility of stereoselective cross-coupling of geminal dibromoalkenes at their *E*-position, but none of those used the remaining bromide for a subsequent coupling step. The group of Yokoyama took advantage of the remaining bromide to perform a second cross-coupling. During their investigations, they managed to perform both coupling steps in a one-pot procedure by simply adding the second boronate, additional base, and raising the temperature after completion of the first cross-coupling (Scheme 25). For less reactive boronates, they also added PdCl_2_(dppf).CH_2_Cl_2_ for the second cross-coupling step [44].

**Figure:**
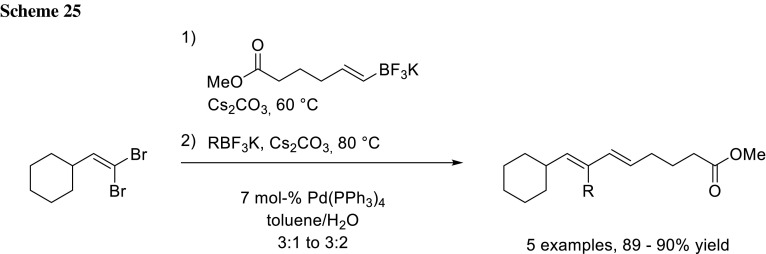

### Selective homocoupling of symmetric arene dihalogenides

In some cases, it is possible to selectively cross-couple one halogenide in symmetric dihalogenides. As in a symmetric substrate, both halogenides have the very same properties in electronical and steric parameters; the resulting product has to be unreactive under the reaction conditions. Otherwise, a mixture of unreacted starting material, the desired mono coupled, and double coupled product will be obtained. There are several ways to achieve selectively mono coupled products. One way is to use the starting dihalogenide in excess. Kelly and coworkers used this strategy to synthesize a library of potent antimitotic agents (**35**) (Scheme 26). Using a twofold excess of their dibromoarenes (**33**), they achieved selective monocoupling [45].

**Figure:**
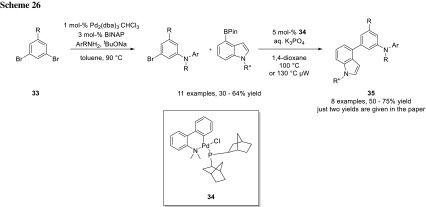

Especially, in longer synthetic sequences with rather complex coupling partners, a large excess of a coupling partner is very wasteful. Using the above strategy is just recommendable using inexpensive commercially available starting materials. The group of Mihovilovic developed a microwave-assisted protocol for selective mono Buchwald-Hartwig amination of 2,6-dichloropyridines (**36**) (Scheme 27) and 3,5-dichloropyridines with a slight excess of amine. They obtained no double amination. The selectivity may be explained in the change of electron-density distribution. The resulting aminochloropyridine is more electron rich than the starting material, making it less reactive for oxidative addition. The remaining chloride can be used for a subsequent Suzuki–Miyaura cross-coupling with Pd(PPh_3_)_4_ (Scheme 27) [46].

**Figure:**
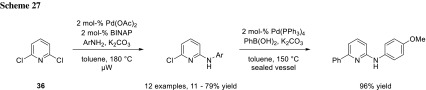

2,6-Dibromopyridine (**37**) was used as a substrate for sequential cross-coupling by Patroniak and her coworkers. After the first cross-coupling step, they subsequently stannylated the remaining bromide. Afterwards, they performed a Stille cross-coupling between the synthesized stannane **38** and **37** (Scheme 28) [47].

**Figure:**
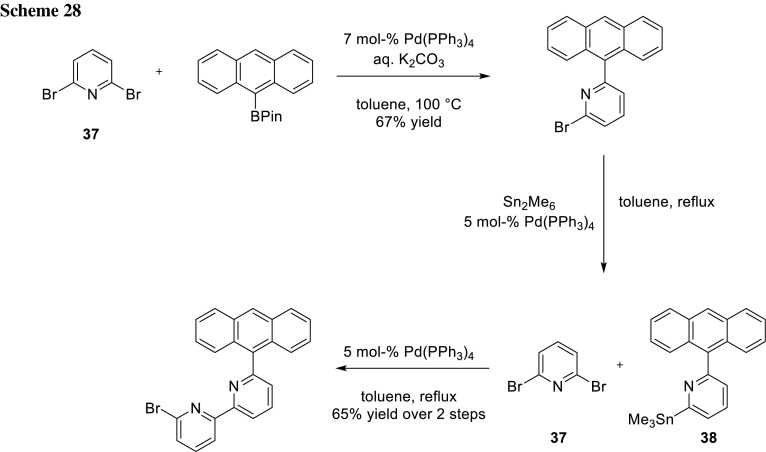

In their total synthesis of leucomelone, Hu’s group used PdCl_2_(dppf) in 1,4-dioxane to achieve a monocoupled benzoquinone in good yields with less than 5% double coupled side-product. The remaining bromide could be cross-coupled subsequently using PdCl_2_(dppf) and CsF in toluene (Scheme 29) [48].

**Figure:**
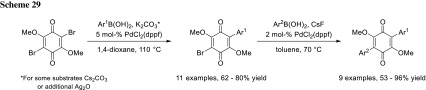

A very interesting way to gain selective mono cross-coupling at 2,6-dichlorobenzaldehyde (**39**) is using PdCl_2_ supported on 4 Å molecular sieves. This system was developed by Ranu’s group and they obtained selective monocoupling even with an excess of boronic acid at elongated reaction times. The authors gave no explanation of this selectivity, but a possible hypothesis would be that the resulting coupling product is too large to reenter the molecular sieves for a second cross-coupling. A subsequent cross-coupling could be achieved using Pd-PEPPSI-*i*Pr as a catalyst (Scheme 30). When Pd-PEPPSI-*i*Pr was used at the first cross-coupling step, the diphenylbenzaldehyde was obtained in one step without any selectivity [49].

**Figure:**
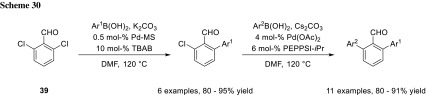

## Regio- and chemoselectivity at the nucleophilic coupling partner

### Masked leaving groups

#### BF_3_K-salts

Masked leaving groups are one way to control the nucleophilic selectivity of cross-coupling reactions. Under certain conditions, these boron species are inert to transmetalation and remain intact during coupling reactions. This characteristic was used by different groups to develop iterative cross-coupling procedures.

Sandrock and Molander synthesized 1,2-dibora species via hydroboration of vinyl-trifluoroborate with 9-BBN [50]. In addition, other nucleophilic fragments can be used containing a trifluoroborate and an olefin for hydroboration [51]. In the absence of water, no fluoride-hydroxyl exchange occurs and the trifluoroborate does not undergo transmetalation, while exclusively the trialkylborane is coupled in THF with KF as base. Switching the solvent to MeOH or toluene/H_2_O and the base to K_2_CO_3_ hydrolyzes the trifluoroborate salt and reveals the free boronic acid which is suitable for transmetalation. A wide range of different products was synthesized using this method in a three-step one-pot procedure tolerating a variety of chloride and bromide electrophiles containing different functional groups as ketones, aldehydes, nitriles, and esters. The scope of the reaction was further explored and the method was also applicable for different kinds of halogenated heterocycles as well as alkenyl bromides (Scheme 31).

**Figure:**
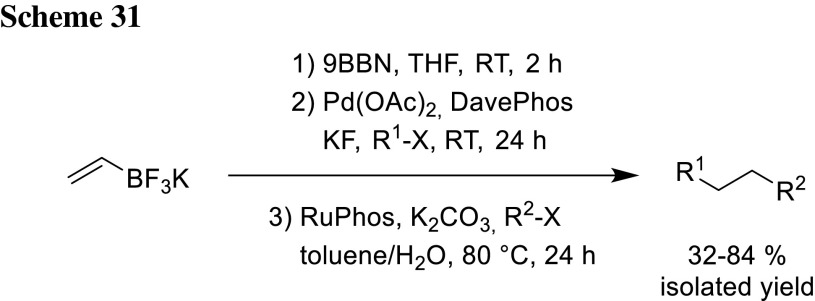

#### Boronic amides

Other boronic acid derivatives can be coupled in the presence of boronic amides due to their reduced Lewis acidity at the boron [52, 53]. Lone-pair donation from the Lewis basic nitrogen makes the boronic amide very unreactive under basic coupling conditions. Among the described boronic amide ligands, 1,8-diaminonaphthyl (DAN) is the most stable, since the lone-pair donation is reduced in anthranilamide (AAM) and 2-(pyrazol-5-yl)aniline (PZA) by carbonyl conjugation and nitrogen aromaticity. The boronic acid is liberated through mild acidic treatment and makes it, therefore, chemically distinct from MIDA boronates which are cleaved under basic conditions. BDAN is introduced by refluxing boronic acids with 1,8-diaminonaphthalene in toluene with azeotropic removal of water, affording the naphthalene-1,8-diamido derivatives in high yields. The corresponding products could be easily purified by either recrystallization or column chromatography (Scheme 32). In addition, the introduction via unsymmetric diboration is possible and regioselective procedures were reported by Sugimone (diboration of alkynes) [54] and Santos (diboration of allenes) [55]. Hydroboration of borylalkenes gives access to 1,1-diborylalkanes as shown by Yun and Hall [56, 57]. Treatment with diluted sulfuric acid or hydrochloric acid at room temperature leads to clean deprotection of the 1,8-diaminonaphthalene group and reveals the free boronic acid.

**Figure:**
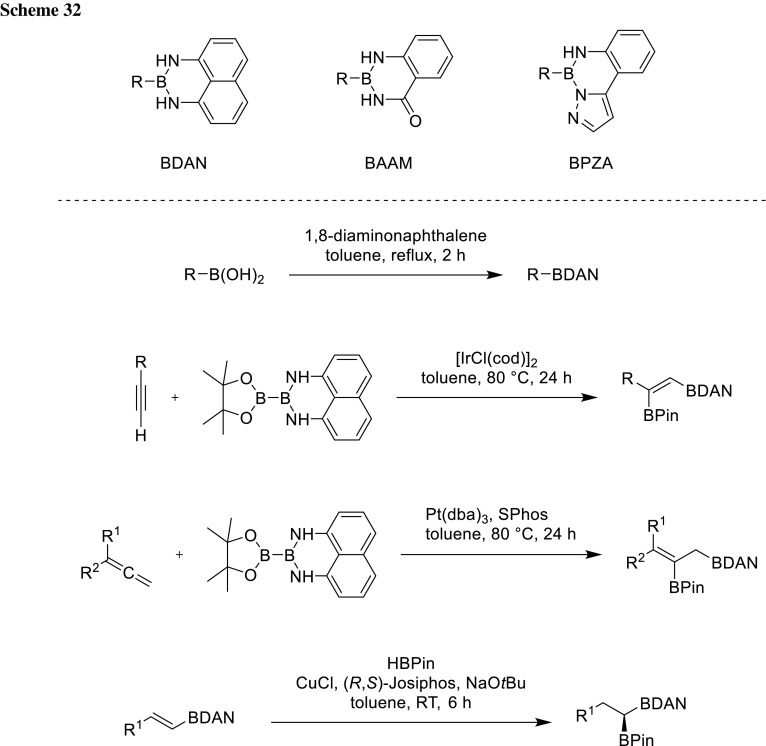

The BDAN protocol was primarily explored by Sugimone and used for the assembly of oligoarenes (**41**) and oligo(phenylenevinylene)s (**43**) from simple bifunctional building blocks [58–60]. The oligoarenes were synthesized from arenes (**40**) containing the electrophile (bromide) as well as the nucleophile (masked boron species). Starting from *p*-tolyl boronic acid, several arene rings can be attached selectively and the last remaining BDAN can be converted to other functional groups (Scheme 33).

**Figure:**
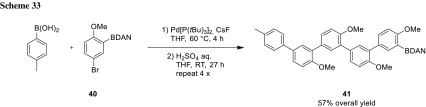

For the synthesis of oligo(phenylenevinylene)s (**43**), BDAN was added to different alkynes via Ir-catalyzed hydroboration, leading to the formation of masked coupling modules (**42**) for iterative cross-coupling. After unmasking the protecting groups, coupling proceeds selectively and gives access to functionalized oligo(phenylenevinylene)s (**43**, Scheme 34).

**Figure:**
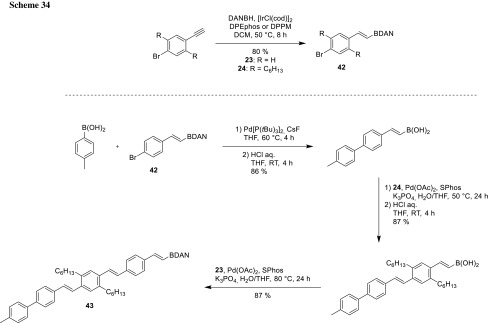

Using Sugimone’s unsymmetric diboration of alkynes, also, a regiocomplementary synthesis of *β*-arylethanols was accomplished. The cross-coupling products derived from the unsymmetric diboration system can be further transformed into 2,2-diarylethanol derivatives via Pd/C-catalyzed hydrogenation and deprotection of DAN followed by H_2_O_2_ oxidation. In contrast, cross-coupling of the symmetric B_2_Ppin_2_-based diboration product provides the regioisomeric alcohol selectively (Scheme 35).

**Figure:**
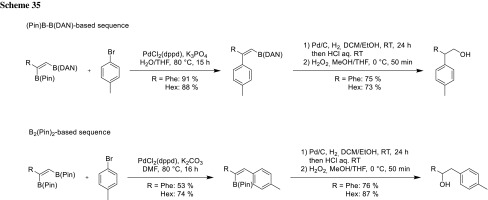

#### MIDA boronates

The most frequently used protecting group strategy was introduced by Burke and uses *N*-methyliminodiacetic acid (MIDA) as ligand. The nitrogen lone pair donates into the vacant p-orbital of the boron leading to a rehybridization from sp^2^ to sp^3^. The Lewis acidity of the boron center is reduced rendering it too electron poor to undergo transmetalation. MIDA boronates are stable to a wide range of common organic transformations, including oxidation/reduction, olefination, Evans-aldol reaction, and halogenation. They tolerate strongly acidic conditions and several coupling protocols, such as Stille, Heck, Suzuki–Miyaura, and Sonogashira [1, 61, 62].

In general, MIDA boronates can be synthesized via the corresponding boronic acid and the MIDA ligand by refluxing in toluene/DMSO under Dean–Stark conditions (Scheme 36) [63]. Burke also published a series of protocols for the preparation of special or sensitive MIDA boronates and a pinene-derived iminodiacetic acid (PIDA) for stereoselective synthesis of boronates [64–70].

**Figure:**
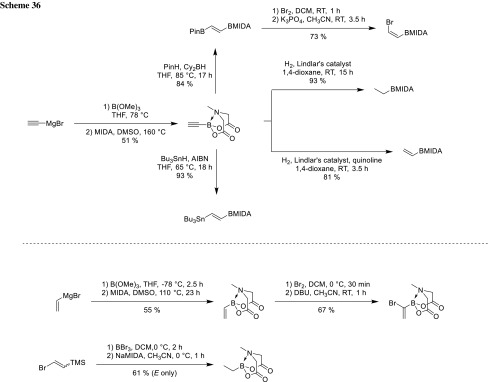

Burke could demonstrate the value of the MIDA boronate system by synthesizing a broad range of various natural products. He retrosynthetically fragmented the compounds in building blocks containing the MIDA boronate and a halogen, and assembled them by similar and repeating coupling as well as deprotecting procedures (Scheme 37) [68, 71–75]. In addition, he was able to set up an automated process for the synthesis of organic molecules using the MIDA protection strategy [76, 77].

**Figure:**
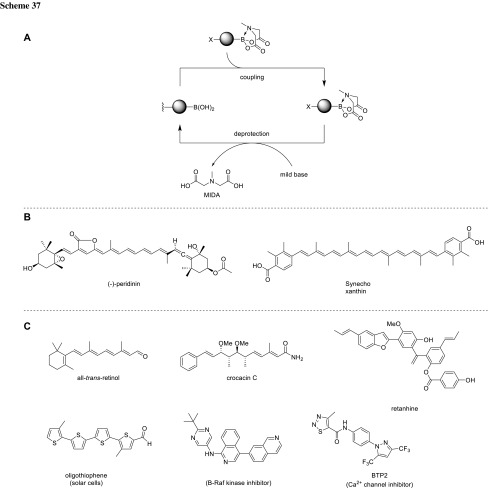

Vosburg also used MIDA protected boronic acids for the synthesis of endiandric-type tetracycles via an iterative cross-coupling procedure. Using this methodology, an inseparable *endo*- and *exo*-diastereomeric mixture of the bicycle could be formed. After forming the bicylic structure, four more steps were necessary to synthesize either a bridged or a fused tetracycle (**44** and **45**, Scheme 38) [78].

**Figure:**
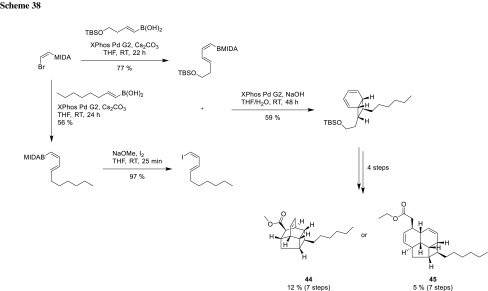

To improve synthetic efficiency protecting group, free methods for controlled transmetalation are desirable. Therefore, Watson developed a protocol for controlled in situ deprotection of MIDA boronates. Accurate chemoselective control of boron solution speciation allows to generate a reactive boronic ester species in situ from the corresponding MIDA boronate. This thermodynamically driven process is strongly temperature dependent and gives access to MIDA boronates at RT or to boronic acid pinacol esters at elevated temperatures. Watson was able to synthesize various boronic acid containing biaryls as well as functionalized phenols where the OH-group is derived from in situ oxidation of the corresponding BPin intermediate (Scheme 39) [79–82].

**Figure:**
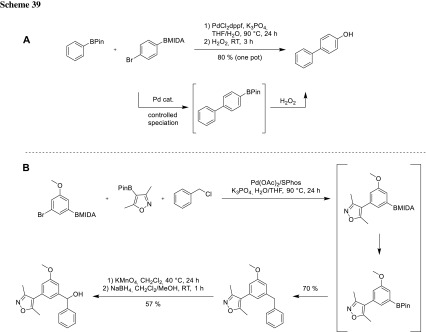

### Chemoselectivity of compounds with two nucleophilic groups differing in their reactivity

Another way to achieve sequential cross-coupling is using nucleophiles of differentiated reactivity. Cook was able to couple MIDA boronates via a combined Suzuki–Hiyama strategy in the presence of silanes to synthesize functionalized stilbenes. After full conversion of the MIDA boronate, the crude product is directly used in the Hiyama coupling. Hydrosilylation gives access to the MIDA–boryl vinylsilanes exclusively in their *E*-configurations (**46**), which remain *E* during the stereo-defined coupling procedure (Scheme 40). These conditions can also be applied for the synthesis of unsymmetrically trisubstituted stilbenes via hydrosilylation of methyl propynyl MIDA ester [83].

**Figure:**
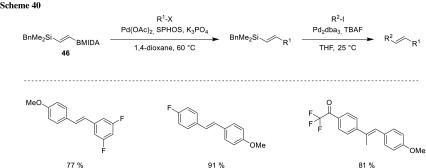

The synthesis of similar BPin-vinylsilanes (**47**) can be achieved by Ru-catalyzed alkene-alkyne coupling as done by Herron (Scheme 41). These molecules undergo the same Suzuki–Hiyama coupling procedure as described by Cook [84].

**Figure:**
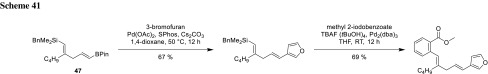

Molander could invert the coupling order using a photoredox and Ni-catalyzed system and primary and secondary ammonium alkylsilicates (**48**) (Scheme 42). Starting with a boronic acid, they observed additional esterification with the catechol from **48** to the catechol boronate, which could be further transformed. Mild reaction conditions and a single electron mechanism allow C(sp^3^)–C(sp^2^) coupling in the presence of different boron species as BPin, BDAN, and MIDA boronates [85].

**Figure:**
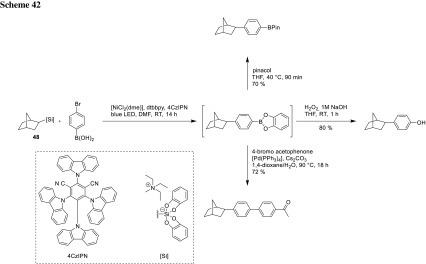

Not only selective coupling of silanes and boronic acid derivatives is possible, but also stannyl groups can be coupled selectively in the presence of different boron species. A chemoselective one-pot Stille–Suzuki cross-coupling reaction of thiophene with aryl bromides and iodides as well as vinyl bromides and alkynyl bromides as electrophiles could be achieved. Since for Stille coupling, no base is needed, the Suzuki coupling can be added to the sequence by adding water and base to the reaction mixture (Scheme 43) [86].

**Figure:**
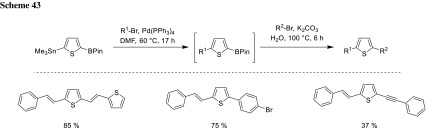

Nishihara could further improve the chemoselective Suzuki/Hiyama coupling by adding an additional Suzuki coupling step, which proceeds regioselectively. The synthesis of a series of geminal (**49**) and vicinal (**50**) diborylated vinylsilanes was accomplished via highly selective Pd-catalyzed *syn*-silylborylation and, respectively, *syn*-diborylation of alkynylmetal species. For the *syn*-silylborylation, highest yields have been achieved in the presence of in situ generated Pd(0)-isonitrile complex, with 1,1,3,3-tetramethylbutyl isonitrile as ligand. All three possible coupling partners can be reacted in a highly chemo- and regioselective manner, whereas the boron species *anti* to the already installed alkyl group is reacting first, followed by the second BPin-moiety. Finally, the silyl group could be coupled after successfully converting it to the corresponding bromide (Scheme 44) [87–89].

**Figure:**
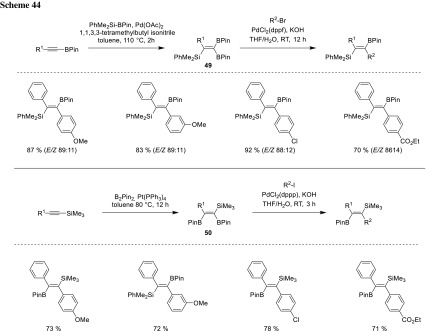

### Regioselectivity control through electronic and steric properties of the substrate

To enable protecting group-free regioselective iterative cross-coupling at room temperature, Shibata used 1,1-diborylalkanes (**51**). The vicinal B atom in 1,1-diborylalkanes seems to influence the boronate moiety as an anion equivalent and can promote the transmetalation between a boronate intermediate and ArPdX. The choice of base seems to be very important, since only strong bases such as MOH lead to the cross-coupling product (Scheme 45). In the presence of K_3_PO_4_, Cs_2_CO_3_, Na_2_CO_3_, and Ag_2_O, no desired product was detected. In addition, the Pd-catalyst seems to be crucial, since Fu´s-related Ni-system was not suitable for this reaction and gave only protodeboronation [90].

**Figure:**
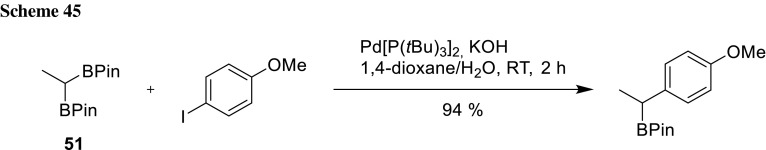

Morken could further explore this reaction and could establish a procedure for enantiotopic group-selective cross-couplings of achiral geminal bis(pinacolboronates). In the presence of a chiral monodentate TADDOL-derived phosphoamidite ligand, the stereoselective synthesis of nonracemic chiral organoboronates with high levels of asymmetric induction is possible. To achieve high selectivity, some critical reaction conditions are important for this synthesis: aryl iodides are significantly more selective than bromides and triflates as well as chlorides did not react at all. Aryl bromides can also be employed as electrophiles, but require the addition of sodium iodide to gain high selectivity. High concentrations of KOH are critical for the reaction and, therefore, a substitution of halide for hydroxide might occur subsequent to oxidative addition. An excess of monodentate ligand is also critical for highest selectivity, since presumably competing background nonligated pathways occur at lower ligand loading as well as in the presence of bidentate phosphines. Morken could demonstrate a broad substrate scope by coupling various aryl halides and geminal bis(boronates), and could also provide a synthesis for (*R*)-tolterodine, a clinically used therapeutic for the treatment of urinary incontinence (Scheme 46) [91].

**Figure:**
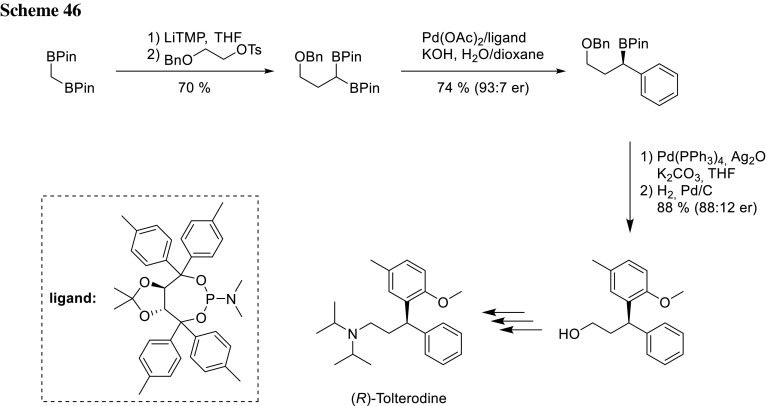

Recently, also, a strategy for asymmetric synthesis and cross-coupling of vicinal alkyl pinacol boronates (**52**) was developed. The first step in this reaction sequence is a Pt-catalyzed enantioselective alkene diboration where chirality is introduced via chiral ligands. After the diboron is installed, the linear boronate undergoes cross-coupling selectively. Morken could show that the 1,2-bis(pinacolboronate)s enhance the rate of transmetalation leading to much higher reactivity of the substrates. He suggested that the adjacent boron atom might act as Lewis acid and coordinates to the pinacolato oxygen. Thereby, Lewis acidity of the neighboring boron center is being enhanced making it more reactive in transmetalation. He could show retention of configuration via labeling experiments, which makes an inner-sphere transmetalation pathway more likely and assisted the Lewis acid activation theory (Scheme 47) [92].

**Figure:**
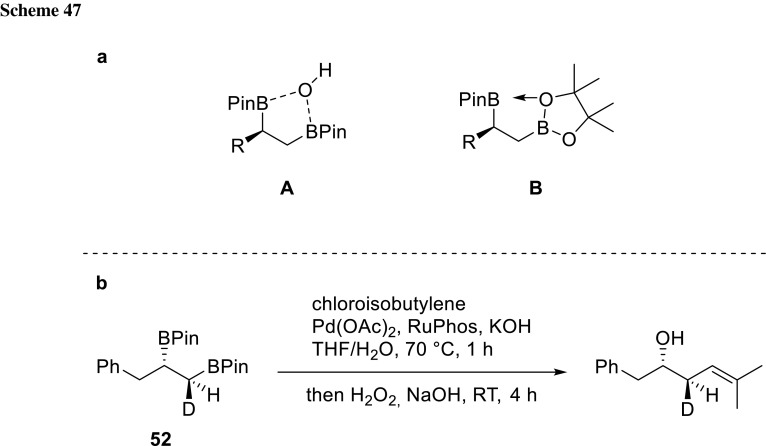

This catalytic enantioselective diboration of terminal alkenes in combination with Pd-catalyzed cross-coupling provides a flexible platform for the construction of a broad array of chiral compounds from nonfunctionalized terminal alkenes and could be applied for natural product synthesis (Scheme 48) [92].

**Figure:**
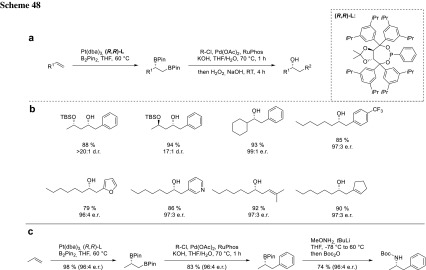

Crudden further exploited the regioselective cross-coupling by taking advantage of inherent differences in the transmetalation efficiency of closely related C-BPin bonds. Orthogonal reactivity of these boronic esters permits iterative cross-coupling without protecting groups at the boron moiety of aromatic, primary, and secondary aliphatic boronic esters. With this method, the Crudden group could synthesize a range of arylated, enantiomerically enriched molecules regio-, as well as stereoselectively and without the need of protection/deprotection steps.

They could perform the first and the second coupling by a two-step one-pot procedure. After a filtration through a small silica plug and the last coupling step, triarylated product **53** could be isolated in 32% overall yield (over 70% per cross-coupling step) (Scheme 49) [93].

**Figure:**
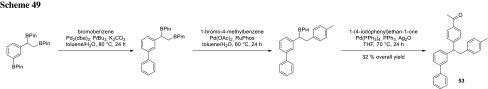

In contrast to the cross-coupling of vicinal boronic acid derivatives where the linear boron species undergoes cross-coupling faster than the secondary boron species, Morken could inverse the reactivity of the boron moieties by adding a directing group to the molecule. He found that a hydroxyl functional group positioned β to a pinacol boronate (**54**) could serve as directing group in Pd-catalyzed cross-coupling reactions. Thereby the β-hydroxy function activates the secondary pinacol boronate and facilitates the cross-coupling reaction (Scheme 50) [94].

**Figure:**
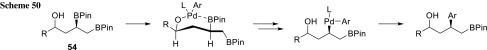

A plausible mechanism for this directing effect might involve binding of the substrate hydroxyl to an LPdAr complex. A subsequent internal delivery of Pd to the nearby secondary BPin would generate an organopalladium complex through an inner-sphere stereoretentive transmetalation and lead to the desired coupling product.

The diborylated starting materials for this transformation can be synthesized via hydroxyl-directed metal-free diboration of homoallylic alcohols (**55**, Scheme 51) [94].

**Figure:**
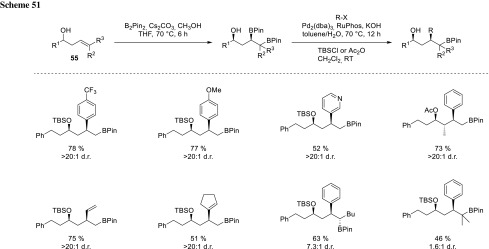

#### Special examples

In 2012, Lassletta used hydrazone as directing group for Ir-catalyzed *o,o*′-diboration of arenes and sequential selective functionalization by Pd-catalyzed cross-coupling (Scheme 52) [95].

**Figure:**
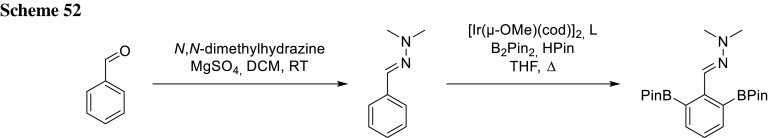

The selective cross-coupling of the *o,o*′-diborylated product is possible based on the dissymmetric interaction of the hydrazone with the two BPin moieties. The hydrazone moiety can form a hydrogen bond to one boronic acid pinacol ester (**56**) and render it more accessible for activation by the base. Therefore, the transmetalation should occur much faster, giving exclusively access to monoarylated products. The second, undesired coupling would require the activation of a more hindered B atom due to electron repulsion between the N-lone pair and the aryl group (Scheme 53) [95].

**Figure:**
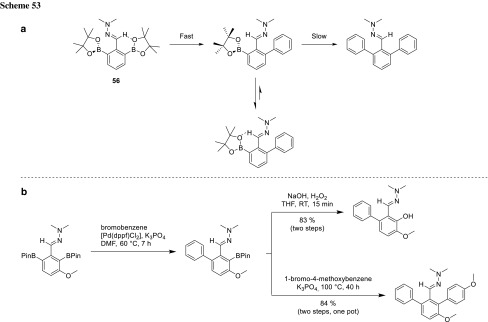

Oestreich developed a side-selective coupling for indoles following a Pt-catalyzed insertion of indolyne into B_2_Pin_2_ via a benzyne intermediate. The 6,7-isomer **57** was the only one coupled in excellent chemo- as well as regioselectivities. Interestingly, the sterically more hindered BPin-group at C-7 underwent cross-coupling first, but the origins of this regioselectivity are still not clarified (Scheme 54) [96].

**Figure:**
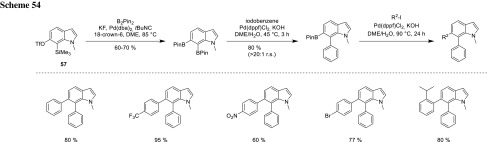

In 2016, Tanaka developed a one-pot, sequential Suzuki–Miyaura coupling using *B*-thexylboracyclanes (**58**). He used a bulky substituent at the boron to ensure endocyclic B-C bond cleavage due to the steric hindrance of the exocyclic B-substituent. The boronic acid subsequently underwent the second cross-coupling under harsher conditions by transfer of the less hindered primary alkyl group to provide the asymmetrically bifunctionalized alkyl chain (Scheme 55). This method is adaptable to 5- to 7-membered rings, whereas the efficiency of the transformation was poorer for 5-membered rings in comparison to the others. Suzuki could successfully prepare several terminally heterobifunctional hexanes in a one-pot reaction [97].

**Figure:**
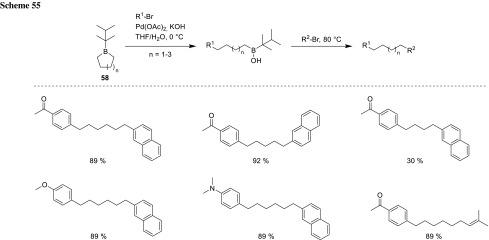

## Selective coupling of reaction partners which can act as nucleophile or as electrophiles

Another set of substrates, which are suitable for sequential cross-coupling, are molecules containing a halogenide/pseudohalogenide as well as an organometallic group suitable for transmetallation. To use these for sequential cross-coupling, reaction conditions have to be developed, at which no oligomerization occurs. The group of Stanetty synthesized thiazoles and oxazoles containing bromide and stannyl (**59**) via a halogen-dance-reaction. With a subsequent Stille cross-coupling reaction, they coupled selectively the substrate at its stannyl-position. Under their reaction conditions the bromostannanes proved to be very unstable, leading to low yields of the desired Stille product with dehydrostannylated substrate as the main product (Scheme 56). They could partly address this problem by performing the stannylation and Stille cross-coupling in a one-pot procedure [6].

**Figure:**
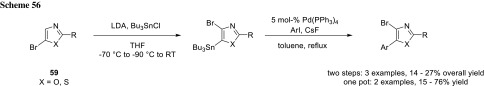

Another example from Narayanawamy’s group shows selective Suzuki–Miyaura cross-coupling between a 2-chloropyridin-4-yl boronic acid (**60**) and 2,4-dichloropyrimidine (**61**). The remaining chloride in the 2-position of the pyrimidine is selectively substituted with MeNH_2_. The last remaining chloride of the pyridine was coupled in a Suzuki–Miyaura cross-coupling. Their example shows a cascade of coupling and substitution events utilizing the different electronic properties of these chlorides. Unfortunately, the authors did not report the yield of the last cross-coupling step (Scheme 57) [98].

**Figure:**
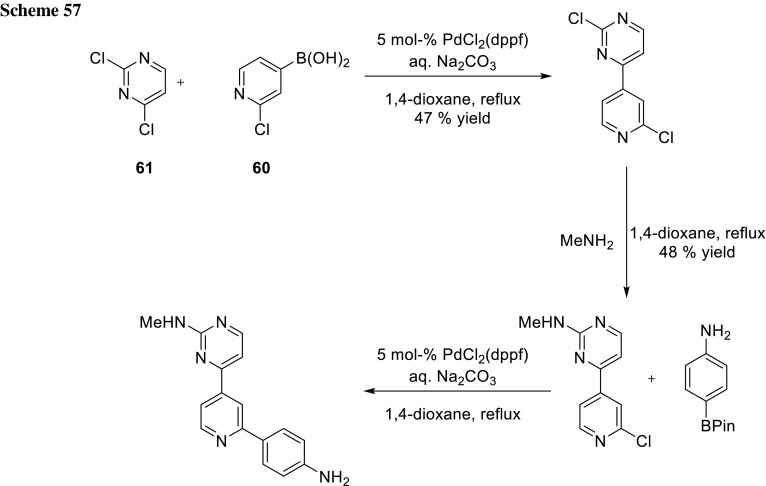

Hall’s group developed a one-pot procedure with 2-iodo-5-methoxyphenylboronic acid (**62**) as substrate. In the first coupling reaction, they selectively coupled the boronic acid of the substrate with an ArI, and by simply adding additional base and a boronic acid, they reacted the electrophilic part of the molecule at the same temperature (Scheme 58) [99].

**Figure:**
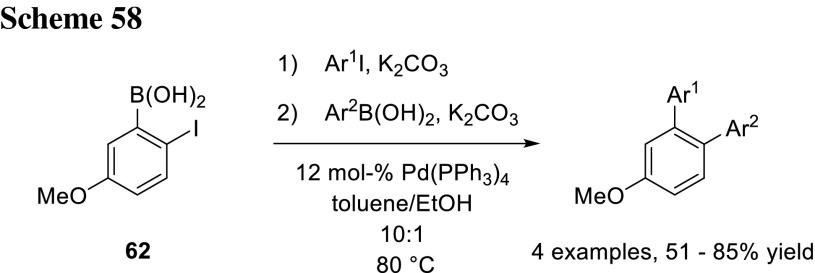

Ray and coworkers utilized 2-bromophenylboronic acid (**63**) in their synthesis of fluoren-9-one derivatives in a sequential-like Suzuki–Miyaura cross-coupling/cyclization sequence (Scheme 59). Using Pd(PPh_3_)_4_ as catalyst with Et_3_N as base in DMF at 90–95 °C, it was also possible to isolate the uncyclizated product in 82% yield [100].

**Figure:**
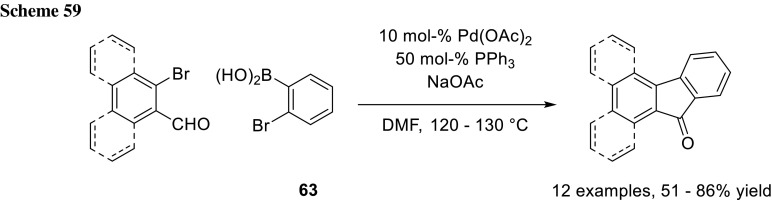

For their total syntheses of canthin-6-one alkaloids, Koutentis’ group utilized substituted 2-chlorophenylboronic acids to react with them first nucleophilically with 8-bromo-1,5-naphthyridin-2(1*H*)-one (**64**). After completion of the first step, they cyclized the coupling product via a C-N coupling by simply adding CuI and DMEDA (Buchwald’s conditions) [101] (Scheme 60) [102].

**Figure:**
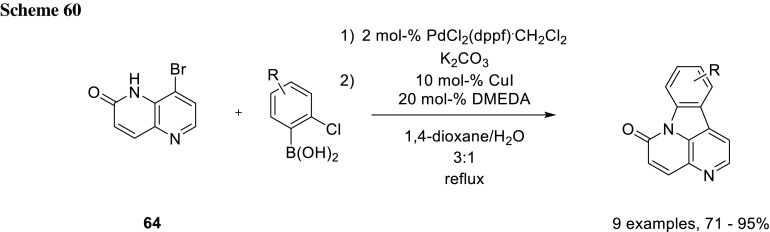

The concept of sequential cross-coupling has even been expanded to be used in solid-phase synthesis by Takahashi’s group [103].

### Suzuki–Miyaura cross-coupling with subsequent CH-activation

Hoarau and coworkers expanded the concept of sequential cross-coupling developing a one-pot procedure with the initial Suzuki–Miyaura cross-coupling with subsequent CH-activation (Scheme 61). Their procedure worked well for their 6-bromoimidazo[1,2-*a*]pyrazine derivatives with various coupling partners. With heteroarylboronic acids as coupling partner, however, their method found its limitation with no cross-coupling product at all [104].

**Figure:**
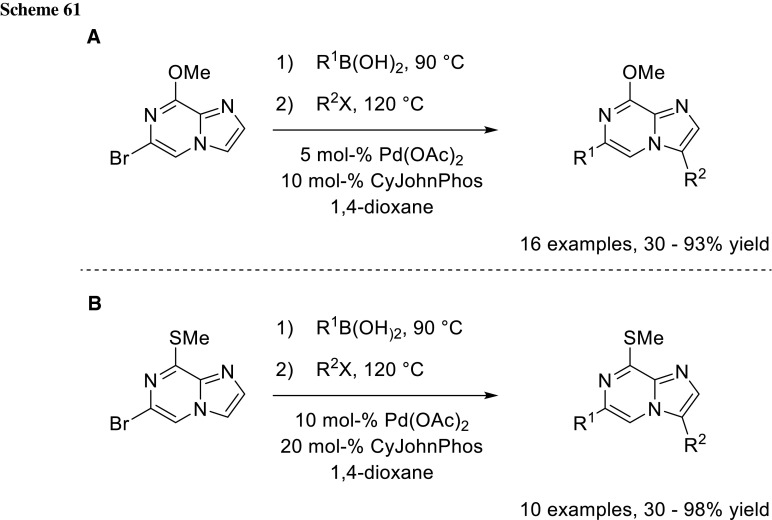

The group of Routier also used this concept to react imidazo[2,1-*b*][1,3,4]thiadiazoles (**65**) by initial Suzuki–Miyaura cross-coupling and subsequent CH-activation in a one-pot procedure (Scheme 62) [105].

**Figure:**
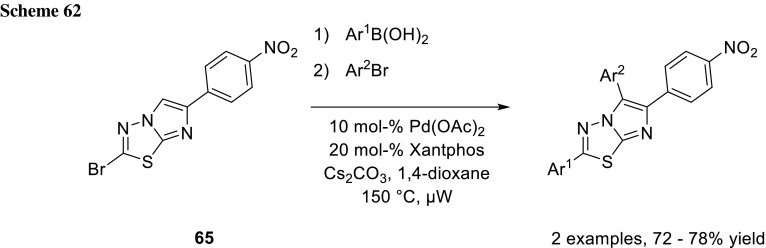

## Conclusion

In recent years, various strategies for sequential cross-coupling have been developed. The selective coupling of the nucleophilic as well as electrophilic site has been achieved, which enabled the exploration of new chemical space. Iterative cross-coupling became an important tool in natural product synthesis and allows the construction of highly complex molecules via simple building blocks. In addition, the concept of sequential cross-coupling enables chemists of creating highly diverse libraries of potential biologically active compounds in a fast and convenient way. Despite these outstanding achievements, there are still many problems not yet solved and worth for further exploration of selective iterative cross-couplings. In particular, the recent progress in CH-activation suggests that the CH bond will be exploited as another attractive leaving group in addition to the well-established halogen and metal leaving groups.
